# Human Intestinal Macrophages Are Involved in the Pathology of Both Ulcerative Colitis and Crohn Disease

**DOI:** 10.1093/ibd/izab029

**Published:** 2021-02-11

**Authors:** Suranga Dharmasiri, Eva M Garrido-Martin, Richard J Harris, Adrian C Bateman, Jane E Collins, J R Fraser Cummings, Tilman Sanchez-Elsner

**Affiliations:** 1 Clinical and Experimental Sciences, Sir Henry Wellcome Laboratories, University of Southampton School of Medicine, Southampton, United Kingdom; 2 University Hospital Southampton NHS FT, Southampton, United Kingdom; 3 H12O-CNIO Lung Cancer Clinical Research Unit, Fundación Investigación Hospital 12 Octubre i+12/CNIO/CIBERONC. Avda Córdoba s/n, Madrid, Spain

**Keywords:** inflammatory bowel disease, intestinal macrophages, Crohn disease, ulcerative colitis, inflammation

## Abstract

**Background:**

Intestinal macrophages are key immune cells in the maintenance of intestinal immune homeostasis and have a role in the pathogenesis of inflammatory bowel disease (IBD). However, the mechanisms by which macrophages exert a pathological influence in both ulcerative colitis (UC) and Crohn disease (CD) are not yet well understood.

**Methods:**

We purified intestinal macrophages from gastrointestinal mucosal biopsies (patients with UC, patients with CD, and healthy donors) and analyzed their transcriptome by RNA sequencing and bioinformatics, confirming results with quantitative polymerase chain reaction and immunohistochemistry.

**Results:**

Compared with those of healthy donors, intestinal macrophages in patients with UC and with CD showed cellular reprograming of 1287 and 840 dysregulated genes, respectively (false discovery rate ≤ 0.1). The UC and CD intestinal macrophages showed an activated M1 inflammatory phenotype and the downregulation of genes engaged in drug/xenobiotic metabolism. Only macrophages from CD showed, concomitant to an M1 phenotype, a significant enrichment in the expression of M2 and fibrotic and granuloma-related genes. For the first time, we showed (and validated by quantitative polymerase chain reaction and immunohistochemistry) that intestinal macrophages in patients with IBD present both M1 and M2 features, as recently described for tumor-associated macrophages, that affect key pathways for IBD pathology, represented by key markers such as MMP12 (fibrosis), CXCL9 (T-cell attraction), and CD40 (T-cell activation).

**Conclusions:**

Our data support the therapeutic targeting of macrophages to maintain remission in IBD but also indicate that a shift toward an M2 program—as proposed by some reports—may not limit the recruitment and activation of T cells because M2 features do not preclude M1 activation in patients with UC or CD and could exacerbate M2-related CD-specific features such as fibrosis and the formation of granulomas.

## INTRODUCTION

Inflammatory bowel disease (IBD) comprises 2 chronic relapsing and remitting inflammatory disorders: Crohn disease (CD) and ulcerative colitis (UC). The etiology of IBD is complex and although not fully understood, it is believed to result from a complex interplay between the host genetics, the microbiome, and an aberrant mucosal immune response.^1^ The interaction of the innate immune system and the gut microbiota in the pathogenesis of IBD seems to be of key importance. This hypothesis is supported by studies that have identified characteristic changes in the gut microbiota of patients with IBD.^2^ Genome-wide association studies and, more recently, exome sequencing have highlighted susceptible loci related to the innate immune system, in particular those involved with microbial recognition and processing such as *NOD2, IRGM,* and *ATG16L1*.^3^

Cells of both the innate and adaptive immune system play a role in IBD pathogenesis. Dendritic cells^4^; B cells^5^; and T helper (th) 1, Th2, Th17, Th9, and Treg^6^ cells have all been implicated in the initiation or propagation of intestinal inflammation in IBD. A major focus of attention has been T cells, leading to discoveries including the development of effective drugs targeting T-cell migration.^7^ However, the intestinal mucosa harbors a range of innate and adaptive immune cells with important immunological roles.

Macrophages are one of the most abundant leukocyte cell types found in the luminal gastrointestinal mucosa.^8^ They are key effector cells of the innate immune system, vital for intestinal mucosal homeostasis.^9^ Animal models of gut inflammation and in vitro studies with human peripheral monocytes have suggested a role for macrophages in the pathogenesis of IBD.^10, 11^ In addition to having a proinflammatory role in the development of intestinal inflammation, macrophages also contribute to wound healing.^12^ Peripheral blood monocyte-derived macrophages from patients with CD have shown impaired bacterial clearance and a reduction in secreted pro-inflammatory cytokines.^9^

Previous studies have suggested a potential benefit for the therapeutic targeting of macrophages in IBD. Some authors have suggested that anti-tumor necrosis factor (TNF)-α therapy may target intestinal macrophages,^13^ resulting in their depletion and moving the macrophage profile from an M1 (inflammatory) to an M2 profile (wound healing).^14, 15^ The M1/M2 classification system of macrophages, initially described as a dichotomy,^16^ has been further developed into a spectrum model of phenotypes with a study by Xue et al,^17^ providing detailed transcriptional profiles of macrophage phenotypes.

We explored the role of intestinal macrophages in healthy patients and patients with UC and with CD to ascertain if some of the clinical characteristics of these diseases can be explained by a pathological function of intestinal macrophages. We decided to use purified populations of intestinal macrophages from patients with well-characterized IBD instead of surrogate models such as blood-derived macrophages or in vivo murine models. We analyzed their whole transcriptome by high-throughput RNA sequencing (RNA-seq). There are limited published data on the application of high-throughput RNA-seq technology in IBD because previous studies have used whole-tissue biopsies rather than isolated specific cell types^18-20^ or, more recently, single-cell RNA-seq of mononuclear phagocytic populations in a very limited number of patients (n = 3).^21^ By isolating intestinal macrophages from a larger patient population, we were able to establish a link between the whole transcriptome of intestinal macrophages and the fundamental clinical characteristics of IBD, including TNF-α-dependent inflammation and T-cell migration, along with fibrosis and granuloma formation in CD. Notably, our data suggest that reprogramming intestinal macrophages in IBD toward an M2 phenotype may not constitute a sound therapeutic approach.

## MATERIALS AND METHODS

### Study Design and Cohort Characteristics

A total of 19 patients with endoscopic evidence of active colonic IBD (9 patients with CD, 10 patients with UC) and 9 healthy control patients were recruited. Regulatory approval was obtained by the Southampton Hospital Research Ethics Committee (Southhampton, UK; reference number 10/H0502/69). Written informed consent was obtained from each patient before the procedure. Colonic pinch biopsies were taken using standard endoscopic biopsy forceps for both healthy control patients and patients with IBD. Samples were taken from the sigmoid colon in patients with UC and healthy donors and from the most inflamed areas in patients with CD. In the patients with CD and with UC, the biopsies were taken from areas of active inflammation; therefore, by definition the patients with IBD recruited to the study all had active disease. Evidence of inflammation in CD/UC was confirmed by a clinical pathologist. For demographic characteristics of the cohort, see Supplementary Tables 1-3.

### Tissue Disaggregation and Macrophage Isolation

Colonic biopsies were immediately processed into single-cell suspensions and preparation for fluorescent activated cell sorting. We obtained 5000 to 10,000 4′,6-diamidino-2-phenylindole glycophorin-A^-^ CD3^-^ CD14^+^ CD163^+^ cells per patient. Previously published single-cell data have suggested that this CD163^+^ population of cells may be enriched for resident intestinal macrophages.^22^ Gating strategy is shown in Supplementary Fig. 1.

### RNA Extraction, Preamplification, and Library Preparation for RNA-Seq

We extracted RNA from the isolated macrophages using the miRNeasy RNA extraction micro kit (Qiagen), following manufacturer instructions. This RNA was preamplified using the SeqPlex RNA Amplification Kit for Whole Transcriptome Amplification (Sigma-Aldrich) to produced amplified dsDNA. From 2 to 10 ng of RNA, the yield obtained was 2 to 3 µg of dsDNA. We generated DNA libraries from the amplified dsDNA using the TruSeq Nano DNA HT library preparation kit (Illumina) in preparation for sequencing.

### RNA-Seq and Data Analysis

For details on RNA-seq and data handling, see the Supplementary Materials and Methods.

### Molecular Pathway Analysis and Gene Set Expression Analysis

Analysis of molecular pathways affected by differentially expressed genes was performed using the Ingenuity Pathway Analysis tool (IPA; Qiagen). Genes filtered by a base mean ≥10, a false discovery rate  ≤ 0.1, and a fold change ≥ |1.5| were loaded into the IPA software.

The Qlucore Omics Explorer 3.2 software package was used for gene set expression analysis (GSEA) analysis. The M1, M2, and granuloma gene sets used for GSEA studies were obtained from Xue et al^17^ (modules 8, 15, and 29) and curated with bibliography. The fibrosis gene set was obtained from IPA software (Qiagen). For more details, see the Supplementary Materials and Methods.

### Real-Time Polymerase Chain Reaction and Immunohistochemistry

For details, see the Supplementary Materials and Methods.

## ETHICAL CONSIDERATIONS

This study was given favorable ethical opinion for conduct in the National Health Service by the Southampton Hospital Research Ethics Committee (reference number 10/H0502/69). All patients who were recruited to the study provided written consent before any involvement in the study. Written consent was obtained according to good clinical practice standards and the protocol approved by the Southampton Hospital Research Ethics Committee (reference number 10/H0502/69).

## RESULTS

### Reprogramming of Transcriptome of Intestinal Macrophages in Patients With Active IBD

**FIGURE 1.**
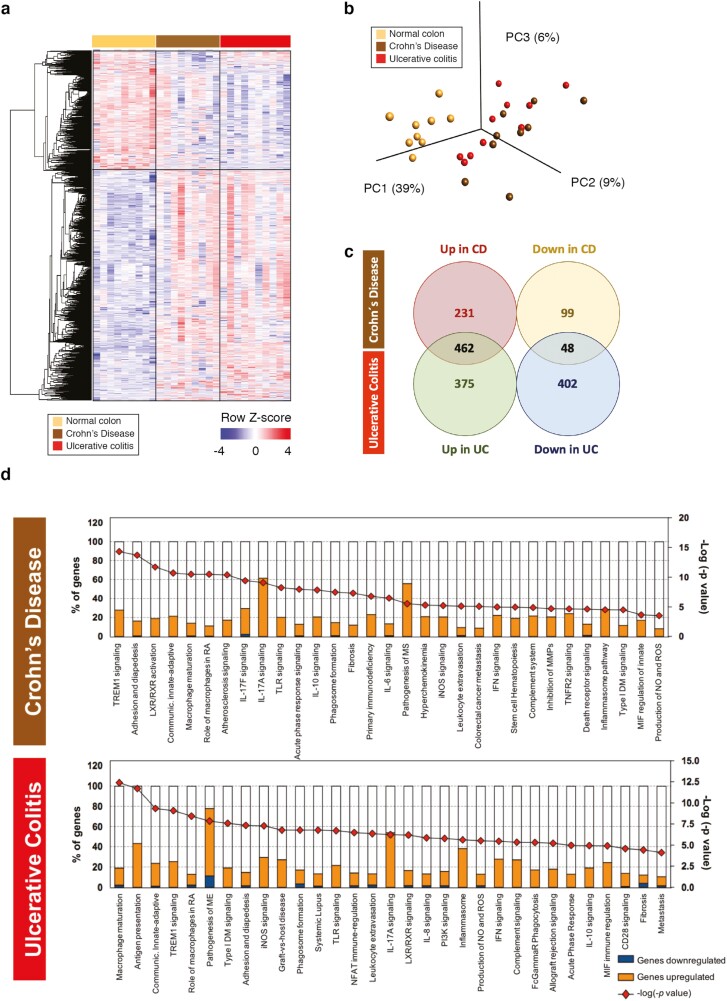
The transcriptome of intestinal macrophages from patients with active IBD is profoundly reprogrammed. A, Heatmap showing differentially expressed genes) found by whole transcriptomic analysis of macrophages isolated from the intestine of patients with IBD (CD, brown, n = 9; UC, red, n = 10) vs that of healthy control patients (normal colon, yellow, n = 9). Pairwise comparison; change in expression ≥|1.5| fold (log2 FC ≥ |0.58|) with adjusted *P* value ≤ 0.1 (FDR; DESeq2 analysis; Benjamini-Hochberg test; mean counts ≥10) presented as row-wise *Z* scores of normalized read counts in IBD macrophages from donors with IBD or healthy donors; each column represents an individual sample and each line represents an individual gene. Number of differentially expressed genes = 1631 with FDR ≤ 0.1 for at least 1 of the comparisons. B, Principal component analysis of intestinal macrophages’ core transcriptomes in patients with IBD and healthy donors. Numbers along perimeter indicate principal components (PC1-PC3) and numbers in parentheses indicate percentage variance for each. C, Venn diagram of the common upregulated and downregulated genes among CD and UC (mean counts ≥ 10; FDR ≤ 0.1; log2 FC ≥ |0.58|). D, IPA showing the signaling pathways mostly activated in intestinal macrophages from patients with CD (up) and patients with UC (down) vs healthy intestinal macrophages. Left axis shows percentage of genes affected per total of genes included in each pathway. Genes upregulated indicated in orange, genes downregulated in blue. Affected pathways ordered from the most statistically significant in decreasing order, where the statistical power of the prediction is shown in red, along the right axis (–log [–*P* value]). Data are from 28 donors (n = 9, normal colon; n = 9, CD; n = 10, UC). Statistical significance baseline corresponds to a *P* value of 0.05. FDR indicates false discovery rate.

We identified a total of 1287 dysregulated genes in intestinal macrophages from patients with UC and 840 dysregulated genes in intestinal macrophages from patients with CD, compared with those from healthy donors. Heatmap representation of the differentially expressed genes showed that intestinal macrophages from UC and CD shared more similarities than with the macrophages purified from the colonic tissue of healthy donors (Fig. 1A). This finding was confirmed by using principal component analysis, in which the samples from healthy donors were in a well-defined isolated cluster compared with those from patients with UC and with CD (Fig. 1B). We found that UC and CD shared just 43% of all the upregulated genes and 8.7% of all the downregulated genes (Fig. 1C), which suggests that intestinal macrophages from UC and CD also have differences between them. We used IPA to assess the functional implications of these observations; IPA identified 171 and 120 canonical pathways, indicating a significant overlap with the identified differentially expressed genes for UC and CD, respectively (*P* < 0.05; Fisher exact test). Interestingly, the top canonical pathways (arranged by *P* value) were largely similar for the UC and CD macrophages (Fig. 1D), indicating a predominantly proinflammatory phenotype (Fig. 1C).

### UC and CD Intestinal Macrophage Expression of M1 and M2 Genes

**FIGURE 2.**
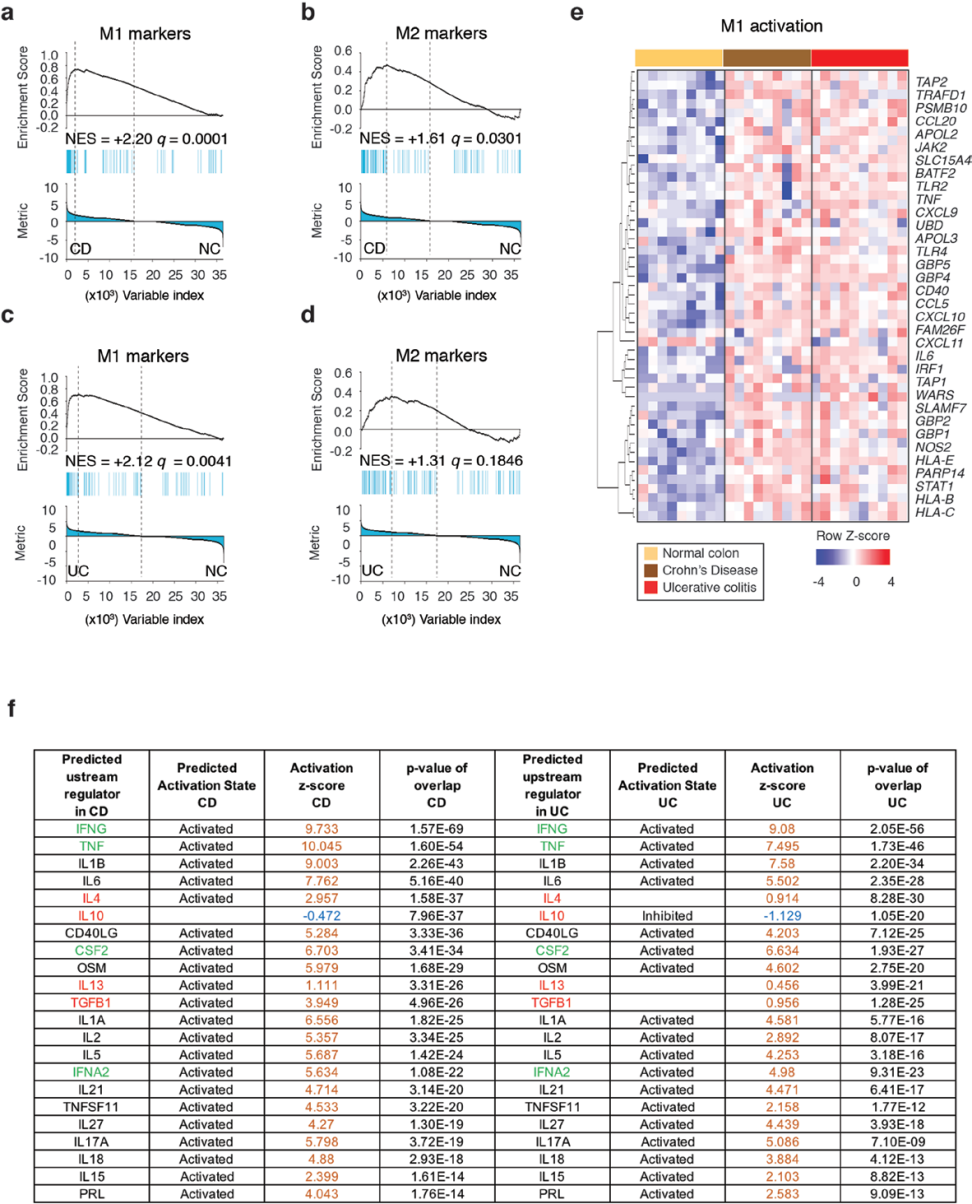
UC and CD intestinal macrophages overexpress M1 genes globally whereas only CD intestinal macrophages show enhanced M2 activation. A-D, GSEA of M1 and M2 gene sets was evaluated in the transcriptome of IBD macrophages vs those of healthy donors. Results presented with Normalized Enrichment Score for the gene set as the analysis moves down the ranked list of genes, reflective of the degree to which the gene set is overrepresented at the top or bottom of the ranked list of genes (top), the position of the gene-set members (blue vertical lines) in the ranked list of genes (middle), and the value of the ranking metric (bottom). *q* values, Kolmogorov-Smirnov test. Data from 1 experiment with 9 donors with CD donors, 10 donors with UC, and 9 healthy donors. A, GSEA of M1 gene set in CD vs NC. B, GSEA of M2 gene set in CD vs NC. C, GSEA of M1 gene set in UC vs NC. D, GSEA of M2 gene set in UC vs NC. E, Heatmap showing M1 activated genes in both patients with CD (brown) and patients with UC (red) vs healthy donors (yellow) with FDR ≤ 0.1 (49 genes). F, Predicted upstream regulators in CD (left) and UC (right) vs those in healthy donors, obtained from IPA ordered from the most statistically significant prediction (lowest *P* value) in descending order (ascending order of *P* value). M1-related upstream regulators indicated in green, and M2 upstream regulators indicated in red. Positive *Z* scores (orange) indicative of activation, and negative *Z* scores (blue) indicative of inhibition. FDR indicates false discovery rate; NC, normal colon.

Macrophages have been loosely defined according to the activation of M1 or M2 phenotypes because these phenotypes are generally linked with Th1 (interferon (IFN)-γ, TNF-α) and Th2 (interleukin (IL)-4, IL-13) immune responses, respectively.^24^ We explored the immune role of intestinal macrophages in CD and UC using GSEA to identify genes known to be associated with M1 and M2 macrophage phenotypes. Macrophages isolated from UC and CD strongly expressed M1 signature genes (Figs. 2A, C, E), which explains why both diseases shared a similar activation of inflammatory pathways such as IL-8, IFN-γ, toll like receptor, TNF-α, and Triggering Receptor Expressed On Myeloid Cells 1 (TREM1) signaling compared with the pathways in healthy donors (Fig. 1). Interestingly, only CD macrophages were significantly associated with the M2 signature genes (Fig. 2D; see heatmap in Supplementary Fig. 2), indicating an important difference between both diseases. Upstream regulators’ analysis by IPA predicted that IL-13 and IL-4 (major M2/Th2 cytokines) and transforming growth factor-β1 (a pleiotropic cytokine linked to fibrosis but also Th2/tolerogenesis) are activated in the CD macrophages but not the UC macrophages (Fig. 2F). Our results indicate that CD and not UC may involve pathogenic activity of IL-4, IL-13 and transforming growth factor-β1 in macrophages.

## ACTIVATED GENE DRIVERS FOR FIBROSIS AND GRANULOMA FORMATION IN CD MACROPHAGES

**FIGURE 3.**
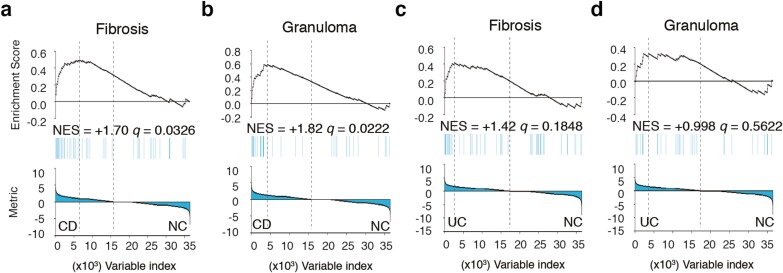
Intestinal macrophages from patients with CD show activated fibrosis and granuloma activities. A-D, GSEA of fibrosis- and granuloma-related gene sets was evaluated in the transcriptome of IBD macrophages vs healthy donor macrophages. A, GSEA of fibrosis gene set in CD vs NC. B, GSEA of granuloma gene set in CD vs NC. C, GSEA of fibrosis gene set in UC vs NC. D, GSEA of granuloma gene set in UC vs NC. The *q* values ≤ 0.05 are considered significant. NC indicates normal colon.

Although we identified transcriptomic similarities between UC and CD intestinal macrophages, we also observed differences in their phenotypes and the predicted upstream regulators controlling their transcriptome. Morphologically, the formation of epithelioid granulomas is found in CD but not in UC. We therefore wondered whether the transcriptome of macrophages could hold clues that would explain these clinical characteristics. We used GSEA to evaluate whether the genes responsible for fibrosis and granuloma formation (see Supplementary Materials and Methods, Supplementary Tables 4, 5) are globally activated in UC and CD. The GSEA showed that the enrichment in the upregulation of fibrotic genes was significant (*q* = 0.0326) in CD but not in UC (*q* = 0.1848) when compared with that of healthy control patients (Figs. 3A-D). Granuloma-related genes were also significantly enriched in the GSEA comparison of CD vs healthy donors (*q* = 0.0222), whereas UC showed no enrichment (*q* = 0.5622). Our results indicate that intestinal macrophages may be linked to fibrotic processes and the formation of epithelioid granulomas in CD but not in UC, suggesting that macrophages could play an important role in these CD-specific pathological features.

## MACROPHAGES IN IBD DOWNREGULATE HOMEOSTATIC AND METABOLIC FUNCTIONS

**FIGURE 4.**
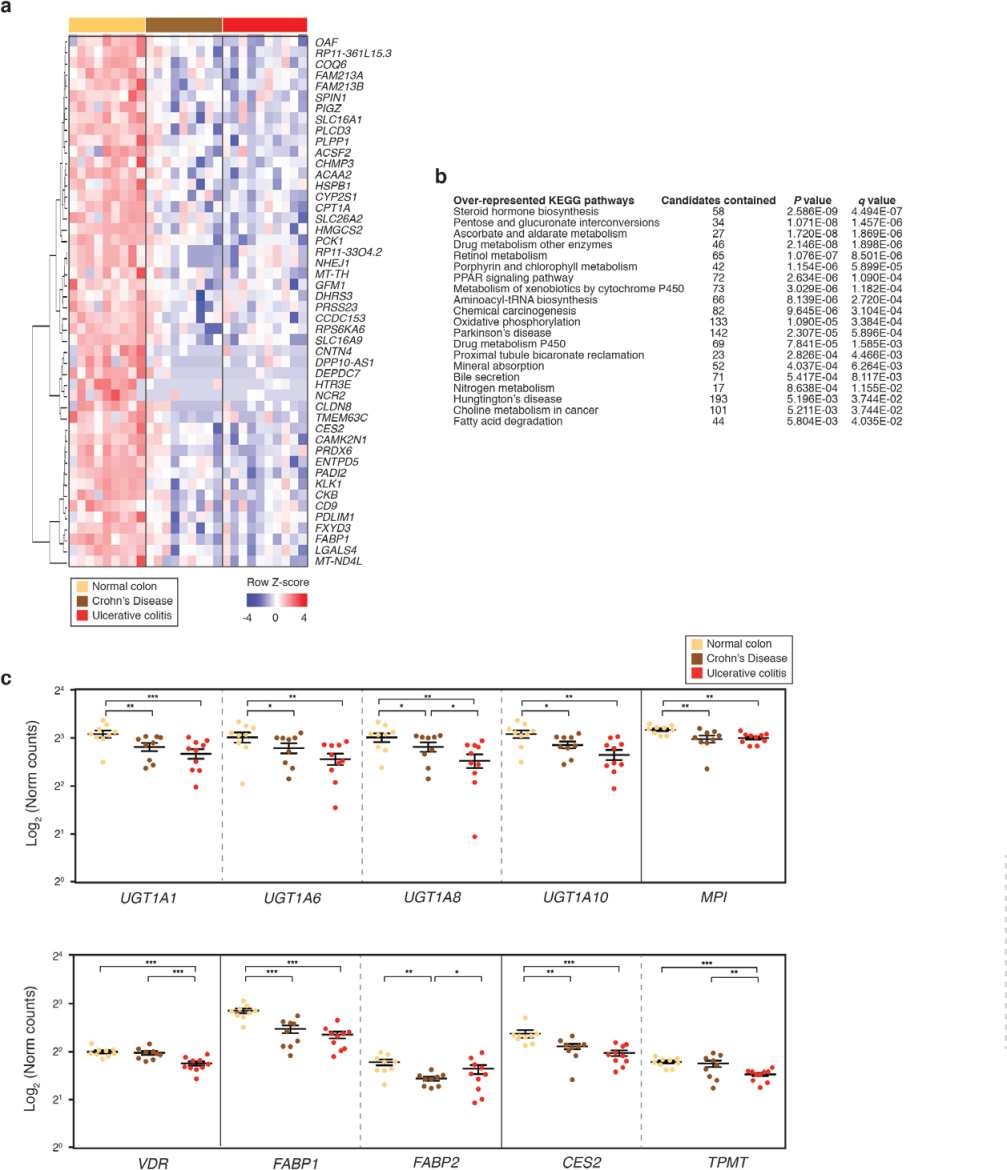
Macrophages from patients with IBD downregulate homeostatic and metabolic functions. A, Heatmap showing downregulated genes in patients with CD (brown) and patients with UC (red) vs healthy donors (NC, yellow). Pairwise comparison; change in expression of 1.5-fold with adjusted *P* value of ≤0.1 (FDR; DESeq2 analysis; Benjamini-Hochberg test; mean counts ≥10) presented as row-wise *Z* scores of normalized read counts. Number of differentially expressed genes = 48 with FDR ≤ 0.1 for at least 1 comparison. B, Overrepresentation analysis (consensus path database) showing Kyoto Encyclopedia of Genes and Genomes pathways enriched in CD and UC vs NC, based on the 48 downregulated genes. C, Downregulated genes related to key homeostatic and metabolic functions. Statistical analysis was performed using Mann-Whitney *U* test. Nonsignificant *P* values > 0.05, *P value ≤ 0.05, **P value ≤ 0.01, ***P value ≤ 0.001. FDR indicates false discovery rate; NC, normal colon.

We have shown that the intestinal macrophages from patients with IBD strongly upregulate inflammatory/M1 activities (in addition to M2, fibrosis, and granuloma functions, only in CD), with potential deleterious pathological consequences . We were interested in whether the overactivation of these inflammatory pathways could impact other cellular functions in IBD. Notably, we found 48 genes downregulated in both CD and UC intestinal macrophages compared with those of healthy donors (fold change ≥ |1.5|, false discovery rate ≤ 0.1, mean Transcripts Per Million counts ≥10). These genes were distributed homogeneously in macrophages from patients with CD and with UC when represented in a heatmap (Fig. 4A). Based on IPA analysis, we observed that the intestinal macrophages in patients with IBD seemed to have important metabolic alterations, specifically in the biosynthesis of steroid hormones, the metabolism of xenobiotics by cytochrome P450, fatty acid degradation, the metabolism of nitrogen, oxidative phosphorylation, and the peroxisome proliferator-activated receptor signaling pathway (Fig. 4B). We discovered a significant defect in glucuronidation, with downregulation in Uridine 5’-diphospho glucuronyltransferases (Fig. 4C), which are involved in pentose and glucoronate interconversions. Glycosidation was also altered, and there was an observed deficiency in mannose phosphate isomerase (Fig. 4C). We also found changes in vitamin D metabolism and the pathways responsible for drug metabolism (*CES2, TPMT*; Fig. 4C). These data suggest that IBD intestinal macrophages stop performing important functions such as protecting the gut from extraneous substances and xenobiotics that may exacerbate the disease or even interfere with some therapeutic effects of drugs.

## DYSREGULATION OF T-CELL ACTIVATION, INFLAMMASOME AND METABOLIC GENES IN IBD MACROPHAGES

**FIGURE 5.**
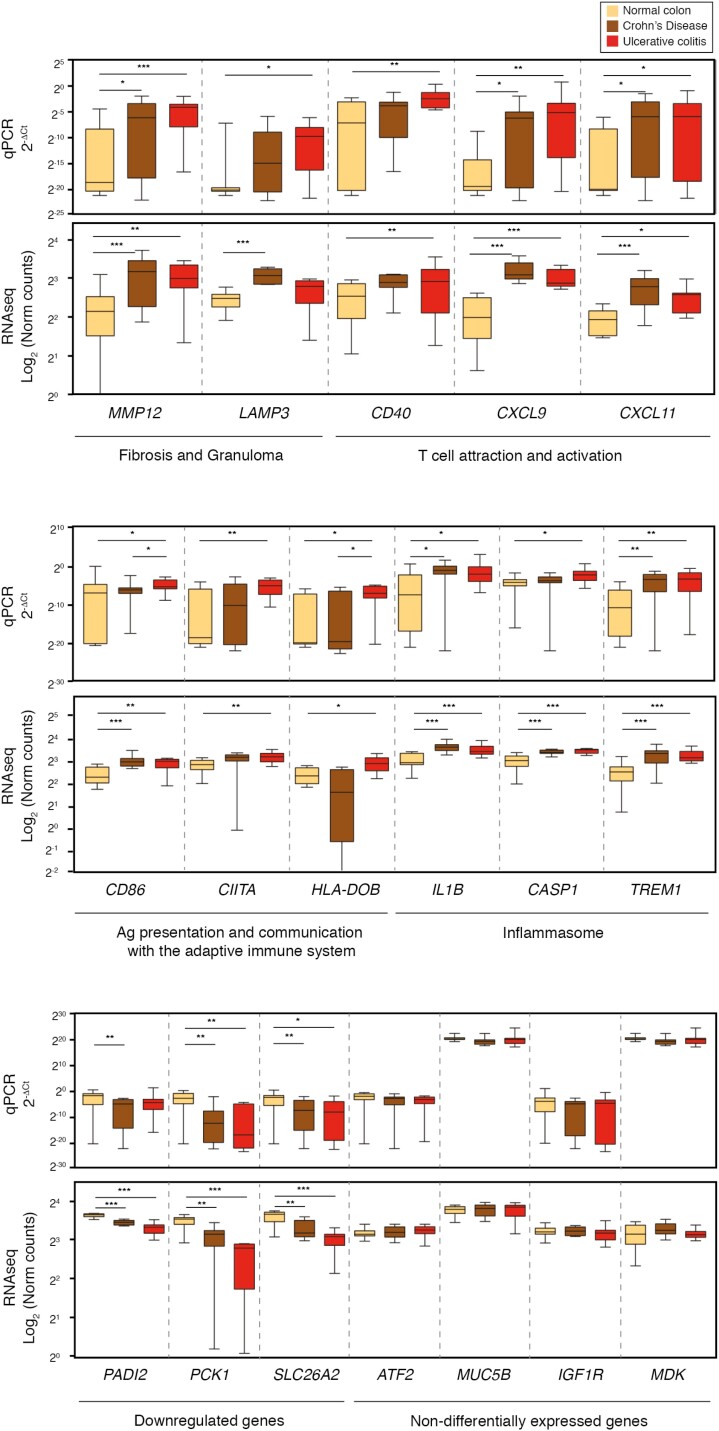
Intestinal macrophages in IBD overexpress genes that attract and activate T cells, upregulate the inflammasome, and downregulate metabolic genes. Validation by real-time qPCR of genes found overactivated or downregulated intestinal macrophages isolated from patients with IBD vs those isolated from healthy patients. Graphs expressed in box plot (minimum to maximum) and error bars represent the standard error of the mean. For qPCR values expressed in 2^-ΔCt^, statistical analysis was performed with the Mann-Whitney *U* test. Nonsignificant *P* value > 0.05, *P value ≤ 0.05, **P value ≤ 0.01, ***P value ≤ 0.001. For RNA-seq values expressed in log2 (normal counts), statistical analysis was explained in the “Materials and Methods” section. Nonsignificant values for FDR > 0.05, *FDR ≤ 0.05, **FDR ≤ 0.01, ***FDR ≤ 0.001. FDR indicates false discovery rate.

We validated the dysregulation of key genes in IBD macrophages using quantitative polymerase chain reaction (qPCR). We confirmed that key inflammatory and immune functions were upregulated in both UC and CD intestinal macrophages: T-cell attraction and activation (CXCL9/11 and CD40), antigen presentation (CD86, CIITA, and HLA-DOB), and inflammasome (IL-1B, CASP1, and TREM1) (Fig. 5). Genes involved in fibrosis and granuloma formation (*MMP12* and *LAMP3,* respectively) also showed differences in expression when compared with these genes in healthy donors. There is strong evidence implicating T cells and T-cell migration to the gut in initiating and perpetuating the intestinal inflammatory process and tissue destruction. An upregulation in antigen-presenting function by macrophages could indicate an increase in the sampling of bacterial antigens from the gut lumen to determine the types of T-cell responses generated.

The inflammasome is a multiprotein intracellular complex that detects pathogenic microorganisms and other causes of cellular stress and activates the highly proinflammatory cytokines IL-1β and IL-18. Inflammasome activation results in cell death by pyroptosis. Research has shown that defective inflammasome signaling by NLR Family Pyrin Domain Containing 3 in the gut contributes to IBD through increased permeability across the epithelial barrier.

Finally, a selection of genes that were downregulated in CD and UC were also validated (*PADI2, PCK1, SCL26A2*), confirming that the metabolism of IBD intestinal macrophages was affected. As a control, we added some genes that showed no change in expression by RNA-seq (*ATF2, MUC5B, IGF1R, MDK*). In summary, we confirmed that IBD intestinal macrophages upregulate proinflammatory functions and have a reduced drug metabolism capacity.

## INFLAMED IBD TISSUE EXPRESSES MORE CD40, CXCL9, AND MMP12 PROTEINS

**FIGURE 6.**
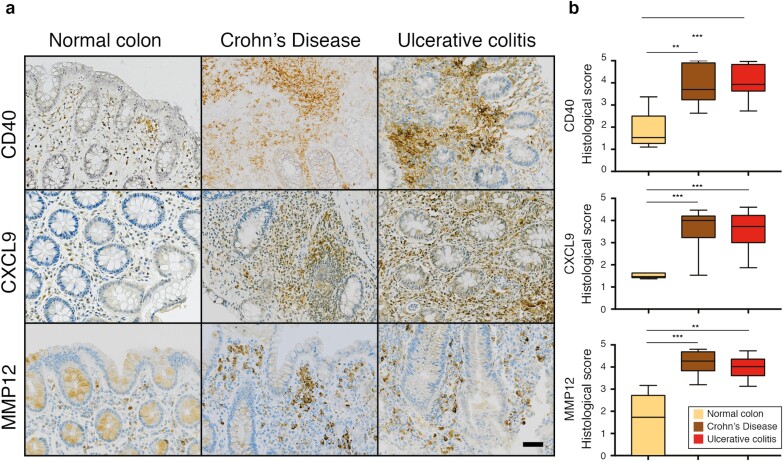
Inflamed IBD tissues express more CD40, CXCL9, and MMP12 proteins. A, Immunohistochemistry showing representative healthy intestinal mucosa from healthy donor and intestinal colonic mucosa from patient with CD and from patient with UC. Staining shows expression of CD40, CXCL9, and MMP12 in 20× magnifications (see 10× magnification pictures in Supplementary Fig. 3). Scale refers to 20 μm. B, Graphs on the right of each image set show quantification of staining in a cohort of patients and donors (CD, n = 13; UC, n = 11; NC n = 5). Statistical analysis performed using Mann-Whitney *U* test. **P value ≤ 0.01, ***P value ≤ 0.001.

Finally, we wanted to assess the dysregulation at the protein level of some of the key differentially expressed genes detected in intestinal macrophages of patients with CD and with UC (already confirmed at the mRNA level by RNA-seq and real-time-qPCR). We chose a protein involved in T-cell recruitment (CXCL9), one involved in T-cell activation (CD40), and MMP12, a typical macrophage protein involved in the remodeling of the extracellular matrix. We performed immunohistochemistry assays in UC, CD, and healthy donor samples. Our results showed that the protein levels of CD40, CXCL9, and MMP12 were significantly increased in patients with IBD when compared with levels in healthy donors, with no significant differences between UC and CD (Figs. 6A, B, Supplementary Fig. 3). This finding confirms the mRNA data in this study and also strengthens the role of macrophages in the gut tissue as key components orchestrating T-cell recruitment and activation and remodeling the extracellular components in the tissue.

## DISCUSSION

Comparison of the intestinal macrophages in patients with IBD with those harvested from healthy control patients indicated a significant change in gene expression in the intestinal macrophages of patients with IBD. This finding allowed us to predict the upstream regulators that may be orchestrating pathology to observe the changes in downstream factors and therefore identify the dysregulated pathways underlying IBD.

The lack of understanding in this area has hampered targeted therapeutic approaches of the disease on an individual patient level. At present, we cannot predict which patients will respond to different therapies or the reasons why some patients respond whereas others do not. It is thus of great importance to understand the pathophysiology of these diseases in individual cell types rather than whole biopsies to develop personalized prognostic and treatment strategies as shown by other IBD studies.^25^

The macrophage population that we selected is enriched for resident intestinal macrophages (CD14^+^/CD163^+^), as suggested by other studies,^21^ although it cannot be ruled out that it contains monocytes recruited to the inflamed mucosa that mature into proinflammatory macrophages,^26^ characterized by CD14^hi^ CD11c^hi^ HLA-DR^dim^ surface expression and the release of proinflammatory cytokines, similar to the ones described in our work.^27, 28^ It seems that CD14^+^ cells are increased in both the noninflamed and inflamed areas of the colon in patients with CD, suggesting that expansion of the CD14^+^ macrophage compartment is not simply a consequence of inflammation, nor does it exclusively result from the infiltration of monocytes.

By combining the multidimensional analysis of the data, not only longitudinal (dataset of gene expression in macrophages) but also transversal (among the different phenotypes—healty donors-CD, healthy donors-UC, and CD-UC), and analyzing them using unsupervised analysis (principal component analysis, heatmap), unsupervised IPA, and supervised GSEA, we obtained an in-depth view of not only the phenotype of macrophages in IBD but also the immune function and metabolic processes that may have therapeutic potential. The activation of pathways such as Nitric Oxide Synthase 2, TREM1, IL-17, and IFN-γ signaling have been previously imputed to the pathogenesis of IBD.^29-31^ Our data may not reflect the full spectrum of the clinical phenotypes of IBD (because of the heterogeneity of IBD), but they are robust and representative of most IBD phenotypes (as shown in Supplementary Tables 1-3), revealing a large number of dysregulated genes in intestinal macrophages from patients with UC and with CD compared with those from healthy donors, resulting in the activation of pathways of inflammation, infiltration of immune cells, antigen presentation, communication between the innate and immune systems, and macrophage maturation.

According to our results, in IBD macrophages there is a derangement of key metabolic homeostatic pathways, which may have significant implications for macrophage function and hence the disease course. This circumstance is well illustrated by the reduced levels of mRNA of genes in the glucuronidation pathway, which is important because the intestinal expression of some UDP-glucuronosyltransferases has been reported as critical for the detoxification of certain chemotherapies such as irinotecan^32^ and thus macrophages could play an important role in pharmacokinetics, which may influence treatment responses in IBD.

Another important metabolic pathway altered is glycosidation: The observed deficiency in mannose phosphate isomerase may be responsible for protein-losing enteropathy.^23^ Our results show that macrophages from patients with UC and with CD have a proinflammatory phenotype. Xue et al^17^ produced an extensive gene expression profile for a variety of macrophage phenotypes from in vitro experiments. We were able to use these expression profiles as a reference for analysis and to phenotype the intestinal macrophages that we isolated from patients with IBD. Notably, one of the pathways enhanced in IBD intestinal macrophages is that of TREM1, a receptor found on macrophages that is responsible for the intracellular amplification of inflammatory signaling.^29^ This condition is consistent with the single-cell RNA-seq results of the Chapuy et al^21^ study, which identified a proinflammatory myeloid group of cells in active CD colonic mucosa that were positive for TREM1. In both mouse models and human ex vivo models of IBD, the blockage of TREM1 seems to dampen the immune response.^33, 34^ Furthermore, the expression of key M1 genes involved in T-cell recruitment and activation (*CXCL9, CXCL10,* and *CXCL11* along with *CD40*) was also increased in IBD macrophages, validated by qPCR and immunohistochemistry (*CXCL9* and *CD40*).

The trafficking of proinflammatory T-cells to the intestinal mucosa is important in the development and propagation of inflammation in IBD,^35^ and our data support macrophages having an important role in this process. Targeting T-cell trafficking has been very successful; vedolizumab, a drug that blocks the α _4_β _7_ integrin/MAdCAM-1 interaction in both CD and UC, is now part of routine clinical practice,^22, 24^ and other drugs such as etrolizumab^36^ are currently being explored in trials. Targeting the secretion of chemokines that promote the migration of T cells to the intestinal mucosa has been explored in seeking new complementary therapies for IBD. So far, there have been mixed results in clinical trials such as blocking the chemokine CCR9^37^ or targeting CXCL10 (using eldelumab) in a phase IIa clinical trial; after promising results in animal studies, the results were inconclusive but suggested a modest benefit in a subgroup of patients.^38^ Our data show that intestinal macrophages in IBD overexpress CXCL9, CXCL10, CXCL11, and other activating molecules, suggesting that manipulating macrophages could be an alternative therapeutic approach to reducing inflammation and infiltration of T cells.

Although the UC and CD macrophages both clearly had an M1 gene expression profile, the CD macrophages also expressed the M2 profile and significantly expressed granuloma and fibrosis phenotypes^17^ (confirmed by GSEA). The dual or mixed M1/M2 phenotype is coherent with recent observations that macrophages can activate both signatures simultaneously.^39^ These findings are consistent with the observed clinical behavior of these diseases, in which fibrotic strictures commonly occur in CD and the presence of granuloma in histological samples can be utilized to differentiate patients with CD from patients with UC.^40^ Future work will determine the role of intestinal macrophages in the formation of fibrotic strictures and granulomas.

## CONCLUSIONS

Our findings add to the accumulating experimental observations that tend to dismiss the dogma that separates macrophages in M1 or M2 phenotypes; our results indicate that M1/M2 labels are not mutually exclusive, not only in tumor-associated macrophages but also in other pathologies, such as IBD. Thus, our key contribution to the future design of future immunotherapies in IBD is that reprogramming intestinal macrophages toward M2 may not only fail to reduce M1 activation (ie, T-cell recruitment and activation) but could also potentially exacerbate fibrotic and granuloma formation in CD; any new drug(s) should be able to tackle and deactivate both M1/M2 pathways, restoring the ability of macrophages to balance the immune homeostasis in the gut lining and contend with xenobiotics.

## Supplementary Material

izab029_suppl_Supplementary_Material

## Notes

Author contribution: Study conception and design was performed by Tilman Sanchez-Elsner, J.R. Fraser Cummings, Suranga Dharmasiri, and Eva M. Garrido-Martin. Material preparation, data collection, and analysis were performed by Suranga Dharmasiri, Eva M. Garrido-Martin, and Richard J. Harris. The manuscript was written by Tilman Sanchez-Elsner and J.R. Fraser Cummings. The writing, review, and editing was performed by Adrian C. Bateman, Jane E. Collins, J.R. Fraser Cummings, and Tilman Sanchez-Elsner. All authors read and approved the final manuscript.

Supported by: E.M.G-M was supported by Cancer Research UK (C30718/A22100 awarded to T.S-E).

## Abbreviations

CD: Crohn disease
GSEA: gene set enrichment analysis
IBD: inflammatory bowel disease
IFN: interferon
IL: interleukin
IPA: Ingenuity Pathway Analysis
PCA: principal component analysis
qPCR: quantitative polymerase chain reaction
RNA-seq: RNA sequencing
Th: T helper cells
TNF: tumor necrosis factor
UC: ulcerative colitis
