# Supplementary material for: Human Intestinal Macrophages Are Involved in the Pathology of Both Ulcerative Colitis and Crohn Disease
Source: Inflamm Bowel Dis. 2021 Feb 11;27(10):1641–52. doi: 10.1093/ibd/izab029 (PMC8522792; doi:10.1093/ibd/izab029)
Supplement: izab029_suppl_Supplementary_Material [file izab029_suppl_supplementary_material.pdf]

Online resources

Electronic Supplementary Materials and Methods

Tissue disaggregation and macrophage isolation

Colonic biopsies harvested at endoscopy were immediately placed in Aqix RSI solution (Aqix) for transportation to the laboratory for urgent processing. The biopsies were disaggregated in an enzyme solution of D-Liberase (Roche) with DNase I (Sigma Aldrich) for 15 minutes at 37°C. Samples were then passed through a 70µm strainer and rinsed 3 times with cold buffer (1xPBS, 2mM EDTA, 0.5% BSA) to form a cell suspension. Cells were then stained for Fluorescent Activated Cell Sorting (FACS) as follows. They were incubated with FcR block (Miltenyi) for 30 mins followed by a cocktail of fluorescent conjugated antibodies for CD45 (HI30, Biolegend), CD14 (MφP9, BD Biosciences), CD3 (SK7, Biolegend), CD8 (SK1, Biolegend), CD163 (RM3/1, Biolegend) and Glycophorin-A (HI264, Biolegend) with an incubation of 30 mins. The cells were further stained with 4'6-Diamidine-2'-phenylindole dihydrochloride (DAPI, Sigma Aldrich) for live/dead staining. We isolated, by cell sorting approximately 5000 to 10000 cells per patient from a cell population that is: DAPI$^-$ Glycophorin-A$^-$ CD3$^-$ CD14$^+$ CD163$^+$.

RNA sequencing

Following quality control for the quality and size distribution of the amplified libraries by chip-based capillary electrophoresis (LabChip, microfluidic system – Caliper Life Sciences) the libraries were loaded onto the flow cell of the Illumina cBot cluster station. The libraries were extended and bridge amplified to create sequence clusters using the Illumina HiSeq PE Cluster Kit v4 and sequenced on an Illumina HiSeq Flow Cell v4 with 100-bp paired-end reads plus index read using the Illumina HiSeq SBS Kit v4.

RNAseq data analysis

The initial processing of the raw RNAseq data was performed by Ocean Ridge Biosciences (ORB) Florida , USA. The raw FASTQ files were spilt into files containing 4,000,000 reads and quality checked using FASTX toolbox [1]. The sequence alignment to the hg38 human reference genome was performed using TopHat v2.1.0 [2] with fr-unstranded as the library type. The Bioconductor easyRNASeq [3] c2.4.7 package running on R version 3.2.2 was used for exon and gene level counting and Ensembl Human Release 83 was used for gene annotation. easyRNASeq settings for read count:

**Setting Name**	**Exon Counting**	**Gene Counting**
Organism	Hsapiens	Hsapiens
Read Length	100L	100L
Annotation Method	RDA file	RDA file
Count	Exons	Genes
Summarisation	-	geneModels

To allow for differential gene expression analysis the gene-level read counts were adjusted for library size and gene length by calculating the reads per kilobase transcript length per million mapped reads (RPKM). The RPKM filter cut off filter used was the 50 read RPKM equivalent for each sample. The 50 read RPKM equivalent value for a given sample was calculated as:

$$RPKM = \frac{\left({}^{50\ reads}/_{2Kbases} \right)}{{}^{\#total\ mapped\ reads}/_{100,000}}$$

Where 2 Kbases is the average length of a gene and the #total mapped reads is the number of uniquely aligned reads from that sample. The average number of reads aligned was 34 million, which corresponded to an average cutoff value of 0.75 RPKM. A gene was therefore considered detectable if its RPKM value was greater than 0.75 in at least one sample. ORB utilise the 50 read RPKM value in their mRNA sequencing pipeline because the RPKM values

of a gene represented by 50 reads should be reproducible in a technical replicates. The 50 read RPKM filter identified 29,403 detectable human genes, the RPKM values of these genes were log2-transformed and used for statistical analysis. The R package DESeq2 [4] was used to analyse the overall effect of Disease (UC - Ulcerative Colitis, CD - Crohn's Disease, NC - Normal Control) on gene expression, and to compare the effects of UC vs NC, CD vs NC, and UC vs CD. Estimated log2 fold changes, which account for the distribution in fold changes across all genes, were calculated in DESeq2 for the same comparisons. All statistical analysis was performed using R version 3.2.2 statistical computing software and DESeq2 version 1.8.1. This data was subsequently used for all differential gene and pathway analysis.

Molecular pathway analysis and Gene set expression analysis (GSEA)

Analysis of molecular pathways affected by Differentially Expressed Genes was performed using Ingenuity Pathway Analysis tool (IPA, Qiagen). Genes filtered by basemean ≥ 10, FDR ≤ 0.1 and Fold Change (FC) ≥ |1.5| were loaded into IPA software. Activated and inhibited cellular functions were analysed by selecting top and bottom Z-scores (Fisher t-test). Upstream regulators were selected only among cytokines and growth factors with the highest activation and highest inhibition prediction using top and bottom Z-scores (Fisher t-test). Canonical pathways were selected by highest p-value and categorised into activated and inhibited by their Z-score (Fisher t-test).

The Gene Set Enrichment Analysis (GSEA) is a computational tool that determines whether an a priori defined set of genes shows statistically significant concordant differences between two biological phenotypes of interest [5]. The Qlucore Omics Explorer 3.2 software package was used for GSEA analysis. GSEA was used to further assess whether specific biological pathways or signatures were significantly enriched between two groups. GSEA determines whether a prior defined ‘set’ of genes (such as a signature) show statistically significant cumulative changes in gene expression between phenotypic subgroups.

RNAseq data public availability

RNAseq data have been deposited on Gene Expression Omnibus under accession number GSE123141 in a private mode. Token for reviewers is irepqsuqdxopziz.

Real time PCR

The cDNA synthesised from the total RNA isolated from colonic macrophages using the RNeasy kit (Qiagen) and pre-amplified using WTA (Sigma Aldrich) was used as a template for real time PCR, performed with TaqMan Universal PCR Master Mix no AmpErase UNG (Applied Biosystems) and TaqMan primers/probes for the genes of interest. GAPDH was used as a housekeeping control. Triplicates for each gene and sample were performed.

Immunohistochemistry

Expression of proteins CXCL9, MMP12 and CD40 was assessed by immunohistochemistry (IHC) using formalin-fixed paraffin-embedded (FFPE) Tissue from gastrointestinal mucosal biopsies from UC, CD and healthy donors. Tissue sections were stained for the chemokine CXCL9 (41906, Novus Biologicals, dilution 1/20), MMP12 (ab137444, Abcam, dilution 1/100) and CD40 (ab13545, Abcam, dilution 1/100). Antibody detection and visualization was performed with low pH heat induced epitope retrieval using EnVision FLEX+ system and DAB as the chromogenic substrate. Optimal antibody conditions were determined in a diagnostic IHC laboratory using automated Dako Link 48 platforms and standardized protocols. Scores for each marker was assessed (1-5 for each slide, 1= weak/absent, 5 = intense staining) by three independent reviewers (blinded to the disease status) and averaged. Pictures were taken on a Zeiss AxioCam MRc5 microscope (Zeiss, Cambridge, UK) and Zeiss Axiovision software (version 4.8.1.0; Zeiss).

1. Lawrence TJ, Kauffman KT, Amrine KC, Carper DL, Lee RS, Becich PJ, et al. FAST: FAST Analysis of Sequences Toolbox. Front Genet. 2015;6:172.

2. Trapnell C, Pachter L, Salzberg SL. TopHat: discovering splice junctions with RNA-Seq. Bioinformatics. 2009;25(9):1105-11.

3. Delhomme N, Padioleau I, Furlong EE, Steinmetz LM. easyRNASeq: a bioconductor package for processing RNA-Seq data. Bioinformatics. 2012;28(19):2532-3.

4. Love MI, Huber W, Anders S. Moderated estimation of fold change and dispersion for RNA-seq data with DESeq2. Genome Biol. 2014;15(12):550.

5. Subramanian A, Tamayo P, Mootha VK, et al. Gene set enrichment analysis: A knowledge-based approach for interpreting genome-wide expression profiles. Proc Natl Acad Sci U S A 2005;102:15545-50.

Electronic Supplementary Figures

Captions

Sup. Fig. S1. **A.** Schematic diagram of the study and laboratory work flow. **B.** Flow cytometry strategy for the isolation of CD14+CD163+ (macrophage) cells. (**a**) Identification of Singlets (**b**) Exclusions of dead cells and red blood cells positively staining for glycophorin or DAPI (**c**) Exclusion of cells positive for CD3 (**d**) Isolation of cells staining positively for both CD14 an CD163. One example is shown for each group (NAC, UC, Crohn's). **C.** Histogram shows the numbers of macrophages FACS-isolated from the colonic biopsies in all three groups. P value >0.9 for all comparison (UC Vs N, UC Vs CD, CD Vs N) (Kruskal-Wallis test).

Sup. Fig. S2. Heatmap showing Differentially Expressed Genes (DEGs) in Crohn's Disease (brown) versus healthy donors (yellow), from M2 activation gene-set (18 genes; mean counts ≥ 10, FDR ≤ 0.1, log2FC ≥ |0.58|). M2 gene-set obtained from Xue. et al and curated with bibliography. Data are from one experiment with n=9 CD donors and n=9 healthy donors.

Sup. Fig. S3. Immunohistochemistry showing a representative case of healthy intestinal mucosa from a healthy subject, intestinal colonic mucosa from a Crohn's Disease patient and from an Ulcerative Colitis patient. Staining shows expression of CD40, CXCL9 and MMP12, in 10X and 20X magnifications. Scale refers to 20 mm.

Electronic Supplementary Tables

Disease activity

All patients with IBD recruited to the study had evidence of active disease demonstrated by mucosal inflammation at endoscopy and therefore by definition were not in remission.

Sup. Table ST1. Ulcerative colitis patients´ demographics

Patient code	Age	Sex	Smoking	Ethnicity	Duration of disease (Days)	Current medication for IBD	Endoscopic MAYO	Full MAYO
UCC535	38	F	N	Caucasian	0	None	1	6
UDC546	69	M	EX	Caucasian	1613	Oral Pentasa, AZA	2	7
UCC583	26	M	N	Caucasian	5222	MXT	2	6
UCC588	44	F	EX	Caucasian	1553	None	2	2
UCC595	76	M	N	Caucasian	1222	Oral prednisolone	2	8
UCC602	24	F	N	Afro-Caribbean	1095	Prednisolone Suppository, Oral Salofalk	1	6
UCC604	39	M	N	Caucasian	0	None	2	6
UDC608	55	F	EX	Caucasian	0	None	2	9
UDC612	39	M	EX	Caucasian	4601	Oral Prednisolone, Oral Pentasa	3	10
UDC613	30	M	N	Caucasian	1686	Oral Salofalk	2	2

Sup. Table ST2. Crohn´s Disease patients´ demographics

Patient Code	Age	Sex	Ethnicity	Smoker	Duration of disease (Days)	Montreal Classification				Previous surgery	IBD Medications	HBI Score
						A	L	B	P			
CDC527	23	M	Caucasian	N	1552	A2	L2	B1	0	N	AZP	7
CCC536	40	M	Caucasian	N	3997	A2	L3	B1	0	N	Oral Prednisolone Oral Pentasa	0
CDC541	35	F	Caucasian	EX	116	A2	L2	B1	0	N	AZP	7
CDC565	66	M	Caucasian	N	5528	A3	L2	B1	0	Appendectomy	None	15
CCC579	45	F	Caucasian	EX	10298	A2	L3	B1	0	N	None	3
CDC594	54	M	Caucasian	N	0	A3	L2	B1	0	N	Oral prednisolone	5
CCC593	49	M	Caucasian	N	4875	A2	L3	B1	0	N	MP	9
CDC606	67	F	Caucasian	Y	1314	A3	L3	B1	0	N	Methotrexate	4
CCC607	49	F	Caucasian	Y	5654	A2	L2	B1	0	N	AZP	5
CDC609	80	F	Caucasian	N	2002	A3	L2	B1	0	N	Methotrexate	8
CDC610	27	M	Caucasian	N	1095	A2	L3	B1	0	N	None	11

Sup. Table ST3. Control subjects´ demographics

Patient Code	Age	Sex	Smoker	Ethnicity	Indication for Endoscopy	Site of biopsy
NAC528	66	M	N	Caucasian	Polyp follow up	Sigmoid colon
NAC564	71	M	N	Caucasian	Polyp follow up	Sigmoid colon
NAC573	74	M	N	Caucasian	Polyp follow up	Sigmoid colon
NAC580	69	M	N	Caucasian	Polyp follow up	Sigmoid colon
NAC597	55	M	N	Caucasian	Polyp follow up	Sigmoid colon
NAC599	60	M	Y	Caucasian	Polyp follow up	Sigmoid colon
NAC601	50	M	Y	Caucasian	Polyp follow up	Sigmoid colon
NAC603	71	F	EX	Caucasian	Polyp follow up	Sigmoid colon
NAC572	78	F	EX	Caucasian	Polyp follow up	Sigmoid colon
NAC605	64	F	N	Caucasian	Iron deficiency anaemia without gastrointestinal symptoms	Sigmoid colon

Sup. Table ST4. – Genes of the Fibrosis module

Genes of the Fibrosis module			
A2M	FGF1	LBP	MYO5A
ACTA2	FGF2	LEP	MYO6
AGT	FGFR1	LEPR	MYO7A
AGTR1	FGFR2	LHX2	NFKB
BAMBI	FN1	LPS	PDGF
BAX	HGF	LY96	PDGFA
BCL2	ICAM1	MET	PDGFB
CCL2	IFNA1	MMP1	PDGFRA
CCL21	IFNA2	MMP13	PDGFRB
CCL5	IFNAR1	MMP2	SERPINE1
CCR5	IFNAR2	MMP9	SMAD2
CCR7	IFNG	MYBPC3	SMAD3
CD14	IFNGR1	MYH1	SMAD4
CD40	IFNGR2	MYH10	SMAD7
CD40L	IGF	MYH11	STAT1
COL1A1	IGF1	MYH13	TGFA
COL1A2	IGF1R	MYH14	TGFBI
COL3A1	IGFBP3	MYH2	TGFBII
CSF1	IGFBP4	MYH3	TGFBR1
CTGF	IGFBP5	MYH4	TGFBR2
CXCL2	IL10	MYH6	TIMP1
CXCL8	IL10RA	MYH7	TIMP2
CXCL9	IL1A	MYH8	TLR4
CXCR3	IL1B	MYH9	TNFA
CYP2E1	IL1R1	MYL2	TNFR
ECE1	IL1RN	MYL3	VCAM1
EDN1	IL4	MYL4	VEGF
EDNRA	IL4R	MYL9	VEGFR
EDNRB	IL6	MYLK	
EGF	FGF3	MYLK2	
EGFR	FGF4	MYO10	
FASL	FGFR3	MYO1A	

Sup. Table ST5. – Genes of the Granuloma module

Genes of the Granuloma module	
ALPK2	IDO2
BIRC3	INSM1
C1ORF115	KTELC1
CASZ1	LAMP3
CD70	LOC727935
CD80	LOR
CMTM6	LYPD3
CSF2RA	MAP3K14
CST7	MCOLN2
EHF	MMP25
ETV3	NAV1
FOXD4	NCCRP1
FOXD4L1	PHF16
GPR64	PYGL
GRASP	RELB

Supplementary Figure S1

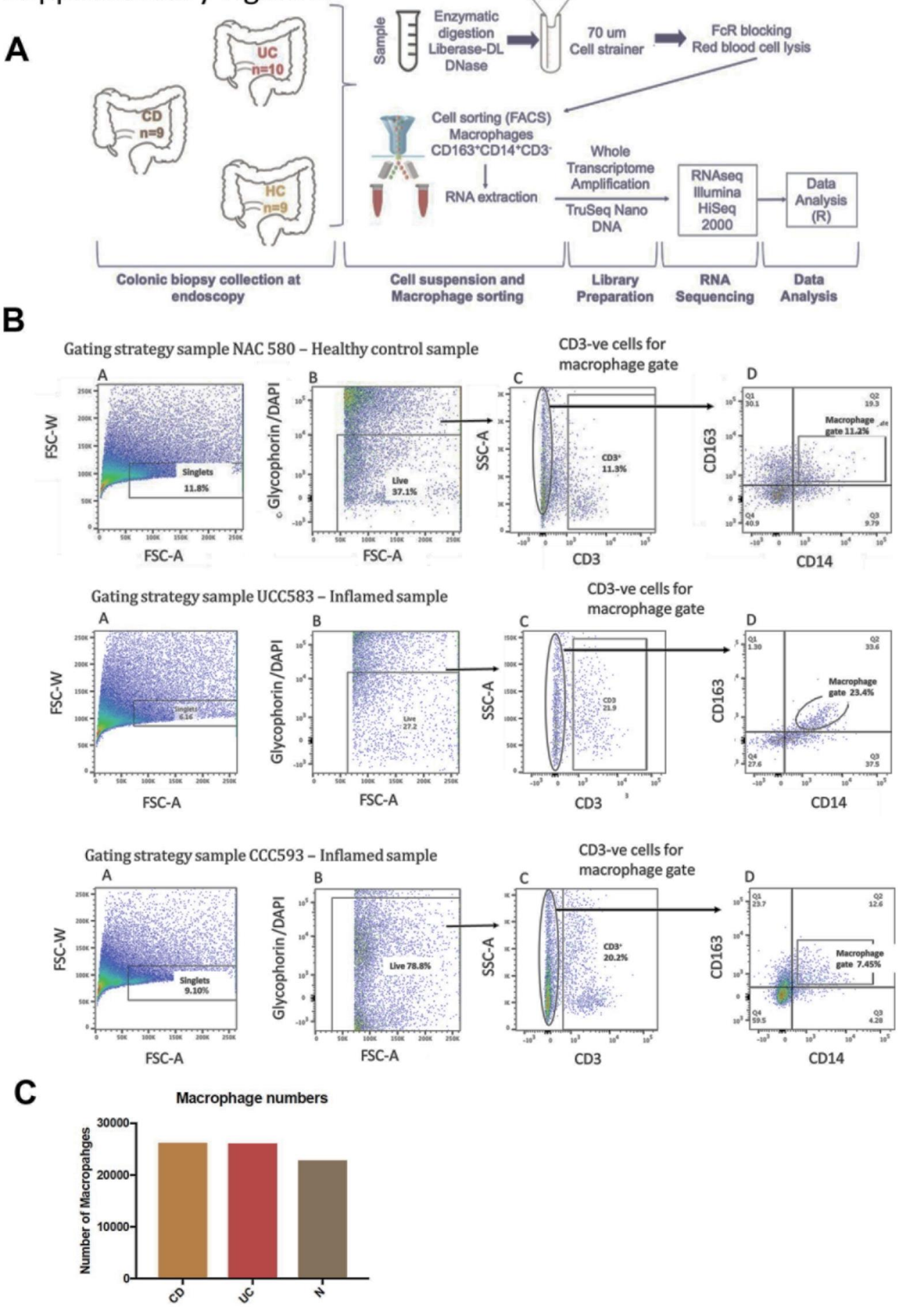

Sup. Fig. S2

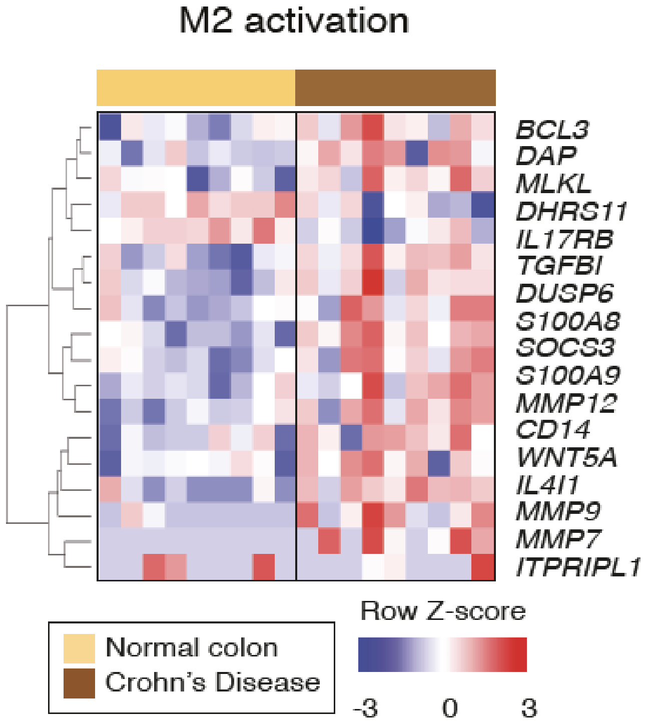

M2 activation
BCL3
DAP
MLKL
DHRS11
IL17RB
TGFBI
DUSP6
S100A8
SOCS3
S100A9
MMP12
CD14
WNT5A
IL4I1
MMP9
MMP7
ITPRIPL1
Normal colon
Crohn's Disease
Row Z-score
-3
0
3

Sup. Fig. S3

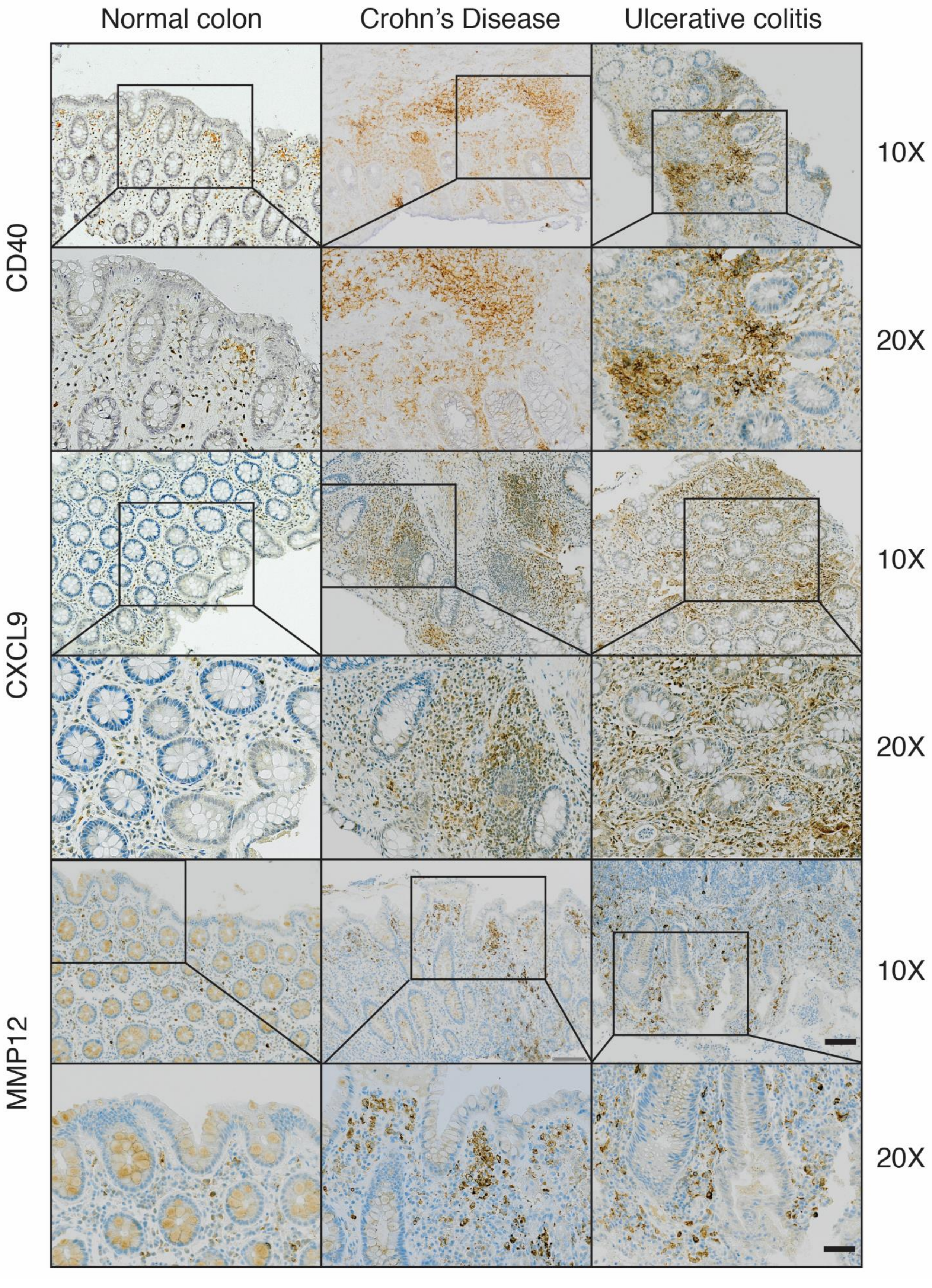
Normal colon
Crohn's Disease
Ulcerative colitis
CD40
CXCL9
MMP12
10X
20X
10X
20X
10X
20X
